# Using Dynamic Features for Automatic Cervical Precancer Detection

**DOI:** 10.3390/diagnostics11040716

**Published:** 2021-04-17

**Authors:** Roser Viñals, Pierre Vassilakos, Mohammad Saeed Rad, Manuela Undurraga, Patrick Petignat, Jean-Philippe Thiran

**Affiliations:** 1 Signal Processing Laboratory (LTS5), École Polytechnique Fédérale de Lausanne (EPFL), 1015 Lausanne, Switzerland; saeed.rad@epfl.ch (M.S.R.); jean-philippe.thiran@epfl.ch (J.-P.T.); 2 Department of Pediatrics, Gynecology and Obstetrics, Geneva University Hospitals, Boulevard de la Cluse 30, 1205 Geneva, Switzerland; manuela.undurraga@hcuge.ch (M.U.); patrick.petignat@hcuge.ch (P.P.)

**Keywords:** cervical cancer, visual inspection with acetic acid, automatic detection, screening

## Abstract

Cervical cancer remains a major public health concern in developing countries due to financial and human resource constraints. Visual inspection with acetic acid (VIA) of the cervix was widely promoted and routinely used as a low-cost primary screening test in low- and middle-income countries. It can be performed by a variety of health workers and the result is immediate. VIA provides a transient whitening effect which appears and disappears differently in precancerous and cancerous lesions, as compared to benign conditions. Colposcopes are often used during VIA to magnify the view of the cervix and allow clinicians to visually assess it. However, this assessment is generally subjective and unreliable even for experienced clinicians. Computer-aided techniques may improve the accuracy of VIA diagnosis and be an important determinant in the promotion of cervical cancer screening. This work proposes a smartphone-based solution that automatically detects cervical precancer from the dynamic features extracted from videos taken during VIA. The proposed solution achieves a sensitivity and specificity of 0.9 and 0.87 respectively, and could be a solution for screening in countries that suffer from the lack of expensive tools such as colposcopes and well-trained clinicians.

## 1. Introduction

In 2020, cervical cancer, a largely avoidable disease, affected approximately 600,000 women worldwide and resulted in more than 340,000 deaths [1]. The World Health Organization (WHO) emphasized the importance of acting immediately to combat cervical cancer through a triple intervention strategy that should be reached by the year 2030, including (i) 90% of girls fully vaccinated by 15 years of age, (ii) 70% of women screened with a high-performance test twice in their lifetime, and (iii) 90% of women identified with cervical disease receive treatment and care.

Cervical cancer is caused by persistent high-risk human papillomavirus (HPV) infection and develops through precursor precancerous lesions named cervical intraepithelial neoplasia grade 2 (CIN2) and grade 3 (CIN3). As far as it concerns screening, WHO recommended, for low- and middle-income countries (LMICs), HPV testing as a primary screening test [2]. Nevertheless, screening with HPV testing alone could result in unnecessary treatment due to its limited positive predictive value and specificity. Therefore, to identify women requiring treatment, visual inspection with acetic acid (VIA) of HPV-positive women is recommended by WHO. VIA has been used for several years in LMICs as a primary screening test, whereby the appearance of aceto-white areas after the application of a 3–5% acetic acid solution helps to define the pathological areas of the cervix and permits their immediate treatment. For countries where HPV testing is not affordable, VIA is still recommended by WHO as a stand-alone screening modality in its “see and treat” strategy [2]. However, VIA is a highly subjective procedure with a diagnostic accuracy for detecting precancer and cancer, varying from setting to setting [3,4,5,6]. Consequently, tools for a reliable noninvasive detection and characterization of neoplastic lesions based on quantitative diagnostic algorithms are desirable to assist front-line providers when performing VIA, especially in low-resource settings.

There are several techniques for computer-aided diagnosis of cervical cancer [7,8,9,10]. Most of them use cytological images [11,12,13,14,15,16,17]. However, an increasing number of studies are developing methods for automatic classification of images captured during VIA, and often, adding images taken during the visual inspection with Lugol’s iodine (VILI) or with the green lens. Particularly, the authors of [18,19,20,21] apply convolutional neural networks (CNN) to images captured with colposcopes.

Nevertheless, standard colposcopes are rarely available for screening in low-income countries due to limited financial resources, health manpower, and facilities. Consequently, recent studies are using other acquisition devices. For instance, a low-cost and portable colposcope has been developed and used to acquire images during VIA and VILI [22,23]. From these images, textural-based features are extracted and used in a support vector machine model. Another example is a colposcope based on a smartphone with optical lenses attached that incorporates an Artificial Intelligence (AI) classifier [24,25]. The smartphone-based system was evaluated in [26], after highly selecting the images and using gynecologic oncologists’ impressions as a reference standard, showing a large variable diagnosis among experts and demonstrating the potential of smartphones as an aiding tool for VIA. In [27], the authors used images taken during VIA with a fixed-focus camera. Finally, authors in [28] developed a digital video colposcope with an optical head that evaluates the effect of aceto-whitening based on the evolution of the diffuse reflectance with respect to time. As a result, it provides a dynamic spectral imaging (DSI) map that has proven to be useful to assist biopsy of suspicious lesions and distinguish high-grade CIN [29,30,31,32].

The approaches described are based on the analysis of a single static image taken during VIA and, often, combining additional images taken during VILI or with the green lens. By contrast, authors in [33] used five colposcopic images taken during VIA and used a graph convolutional network to classify them. Similarly, authors in [34] used multiple images taken during VIA, one image during VILI, and one taken with the green colposcope lens.

In this project, instead of using a colposcope and static images, we use dynamic images recorded via a smartphone during VIA [35]. It is our conviction that the different evolution of the color in the images of neoplastic and healthy tissue after application of acetic acid is a key element for detecting cervical precancer and cancer. Furthermore, the minimal training required for using our diagnosis system, and the increasing penetration rate of smartphones in LMICs, makes it ideal for countries with limited healthcare resources, where it can be easily integrated into a single-visit approach [36].

The rest of the paper is organized as follows. Section 2 presents the dataset and the proposed classification algorithm. In Section 3, the performance of the proposed scheme is analyzed by comparing it with the histology results, the colposcopists’ diagnosis, and the colposcopists’ annotations after VIA. Finally, Section 4 presents an evaluation of the results and Section 5 concludes the paper.

## 2. Materials and Methods

### 2.1. Dynamic Image Dataset

The appearance of aceto-white areas after the application of an acetic acid solution helps to define the pathological areas of the cervix. The CIN2 and CIN3 precancerous lesions (collectively referred to as CIN2+) tend to become whiter just after the application of the acetic acid, and then the whiteness decreases smoothly. By contrast, in the non-neoplastic tissue, the whiteness remains more constant. Most lesions can be distinguished one minute after the application of acetic acid, although it is reasonable to do VIA up to 3 min [37]. In this work, the temporal evolution of neoplastic and non-neoplastic areas will be the key feature used for its classification.

#### 2.1.1. Smartphone as an Acquisition Device

To acquire the dynamic images, we use a Samsung Galaxy S5 (Samsung, Seoul, South Korea) with a camera of 16 megapixels, that record 2 min videos during VIA at 1 frame per second (fps). Patients are placed in the lithotomy position and a speculum is inserted and adjusted to provide more visibility. The smartphone is fixed to a tripod to minimize the movements during recording and placed around 15 cm of the vaginal vestibule. The recording is started immediately after the application of 3% acetic acid.

Our smartphone-based artificial intelligence classifier is intended to be introduced in LMICs using primary HPV screening in a “screen and treat” approach. The mobile app will be used as a triaging method to refer women testing positive for HPV for treatment. Therefore, our dataset used to train the algorithm is derived from a selected group of HPV-positive women aged between 30 to 49 years, which is the age group recommended by WHO for screening asymptomatic women in LMICs [2].

Video sequences were obtained after informed and signed consent from 44 asymptomatic HPV-positive women at the colposcopy consultation in Maternity of Geneva (Geneva University Hospitals), Switzerland, and at the Gynecologic Department of the District Hospital of Dschang, Cameroon. The cases from Geneva were referred for colposcopy after positive cytology and HPV testing, whereas Cameroonian cases were women screened with HPV testing in a “test-triage-and-treat program”. All HPV-positive women received VIA for triage and biopsies, endocervical brushing (ECB), and cytology as a quality control measure [36].

Cervical tissue obtained from biopsies, ECB, or conization served as the reference standard to evaluate the accuracy of the algorithm in this study. Adjudicated histopathology diagnosis was made after preparation of slides in the Division of Pathology (Geneva University Hospitals). Guided biopsies were performed on all visible lesions. If no lesion was seen, a random biopsy at 6 o’clock within the transformation zone and near the squamocolumnar junction was obtained. ECB was performed on all women, with or without visible lesions. The adjudicated diagnoses of 44 cases revealed 15 of them as negative (including 3 CIN1 cases) and 29 as positive (11 CIN2 and 18 CIN3 cases).

Three expert colposcopists (gynecologic oncologists) from the Geneva University Hospitals analyzed the 44 recorded videos, blinded from the histopathologic diagnoses, and were asked to (i) classify them as positive (CIN2+) or negative and (ii) draw the neoplastic lesions on the last frames of the videos.

Note that more sequences had been acquired but some of them were discarded due to severe movement and blurriness. The dataset specifications are shown in Table 1. Due to the limited amount of data, the classification is performed pixel-wise, classifying each pixel independently. Leave-one-out cross-validation was applied at the patient level, i.e., the video’s pixels of the patient being diagnosed were not used to train the model for that patient.

#### 2.1.2. Labels

Each acquisition is labelled negative or positive based on histologic assessment, and, when positive, the neoplastic area (CIN2+) is delineated manually in the last frame of the dynamic image by an expert colposcopist. Two challenges are faced here. First, the selected area may be inaccurate for the pixels close to the boundaries. Second, movement during acquisitions causes variations of the acetowhite region’s position from frame to frame.

**Figure 1:**
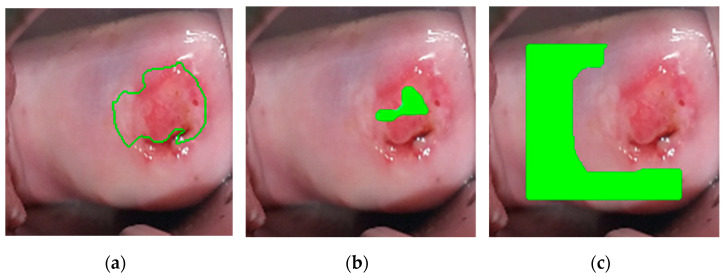
For the supervised learning, positive and negative pixels are chosen from area far from ground truth contours, to avoid mislabeled training data due to inaccurate labeling. From left to right: (**a**) Label drawn by the colposcopist, validated by biopsy, (**b**) eroded label, where positive pixels are chosen from (green region), (**c**) dilated and masked label, where negative pixels are chosen from (green region).

To palliate these two issues, the ground truth labels are eroded and dilated using a disk as a morphological operator whose size is manually set based on the size of the positive region. Pixels for training the neural network are randomly selected from the modified labels: positive pixels from the eroded label and negative pixels from the dilated, ensuring that none of the pixels selected are close to the boundaries between neoplastic (CIN2+) and non-neoplastic regions and the image borders. Figure 1 shows a label drawn by a colposcopist after histological confirmation of a CIN2+ lesion, and the resulting eroded and dilated labels. Note that the area where negative pixels are chosen was obtained not only by dilating the original label but also masking it to avoid regions far from the center of the cervix. From the 44 recorded videos, 21,851 positive pixels and 93,725 negative pixels were selected for training.

### 2.2. Classifier

As mentioned previously, this work is based on the analysis of dynamic image sequences of the cervix under a contrast agent to observe the reaction of cells with acetic acid (VIA) during a defined time window, and regarding the color evolution of each region, deciding whether that region is suspicious or not. Therefore, the intensity curves of the pixels of the video are the key elements of our prediction.

**Figure 2:**
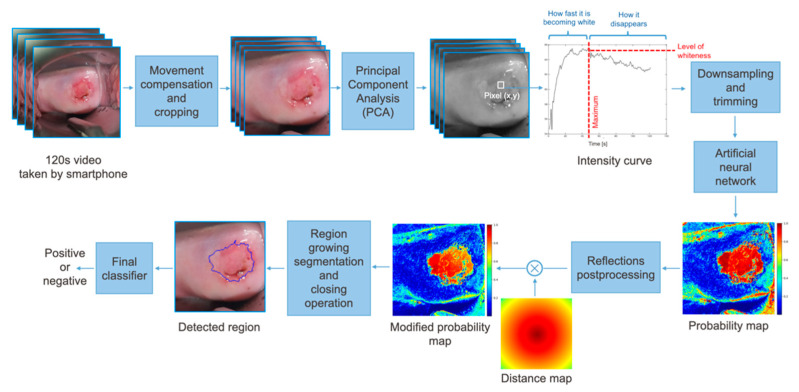
Pipeline of the proposed approach. The features mentioned on the intensity curve, such as how fast it is becoming fast and level of whiteness, are only human interpretation of the curves and are not pre-computed; the proposed network only uses the raw data at its input.

To do so, as shown in Figure 2, we propose a pipeline to process each dynamic image. The different components of the pipeline are described in this section.

#### 2.2.1. Preprocessing

**Figure 3:**
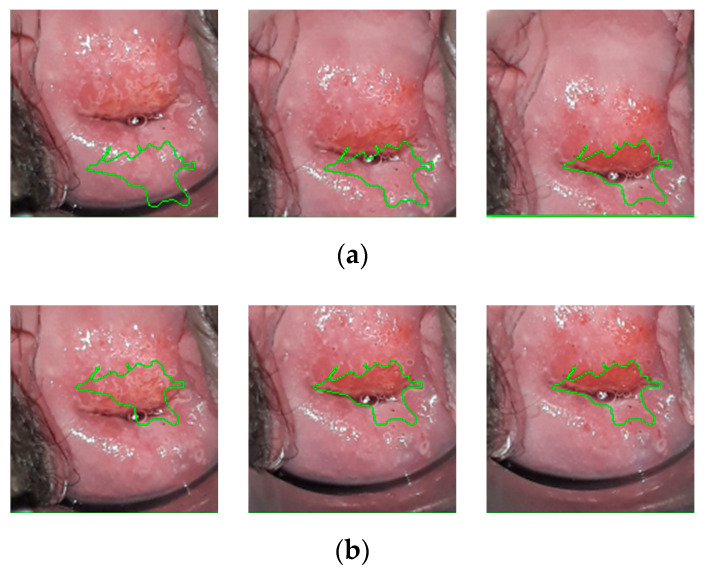
(**a**) Ground truth labels drawn on the first, one-minute, and last frames from a recording before motion compensation. (**b**) Ground truth labels drawn on the first, one-minute, and last frames from a recording after motion compensation.

From each patient, a two minute Red Green Blue (RGB) video was captured, and it was sampled to obtain 120 frames. Motion compensation based on the point feature matching technique [38] was applied, ensuring the method to be robust to small movements of the camera. The frames are cropped to center the cervix and spatially resized to have a resolution of 150 × 150 pixels. An example of the effect of motion compensation is shown in Figure 3 where the ground truth labels, drawn by the expert colposcopist in the last frame of the video, are plotted in the first, middle, and last frames of the recording. When considering the frames recorded without applying motion compensation, the region bounded by the colposcopist’s label changes considerably from frame-to-frame, while when stabilizing the video, the defined region remains more constant.

**Figure 4:**
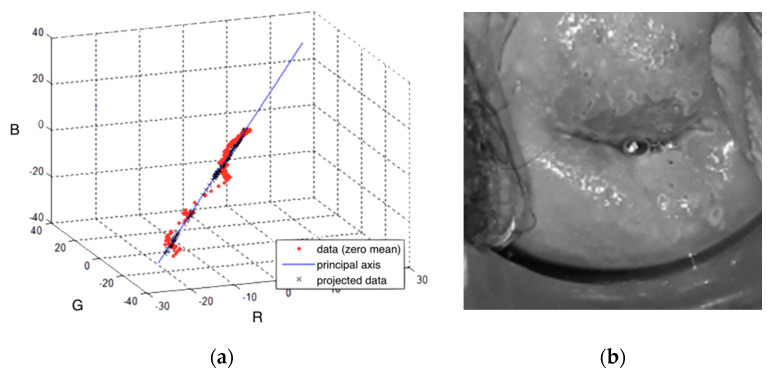
(**a**) An example of Red Green Blue (RGB) values of a positive pixel, projected on the principal axis, to reduce the dimensionality of the input data. (**b**) Resulting video frame after combining the three color channels.

After stabilizing the video, each color channel was scaled to the range [−0.5, 0.5]. To reduce the complexity of the problem, we used principal component analysis (PCA) [39]. PCA is defined as an orthogonal linear transformation that transforms the data, an RGB vector in this case, to a new coordinate system with a reduced dimensionality. The RGB values of a given positive pixel for each frame of the video create an elongated cluster which can be represented by a line. This line, called principal axis and represented in Figure 4a, is estimated to keep the variance at its maximum. Once this axis is obtained, the input data is projected to convert a three-channel pixel to a scalar, while keeping the color evolution still visible (Figure 4b). Note that the principal axis is not the same for every sequence. However, as their variances are not significant, we used the average line of principal axes of our training set.

Once the RGB pixel values are converted to a scalar, the first ten frames are discarded since they are the most affected by movement. The sequence is then downsampled to 11 time points using cubic spline interpolation, which are the final input to the neural net. Recall that the classification is performed pixel-wise. Thus, each pixel with its corresponding 11 time points is an independent input to the neural network.

**Figure 5:**
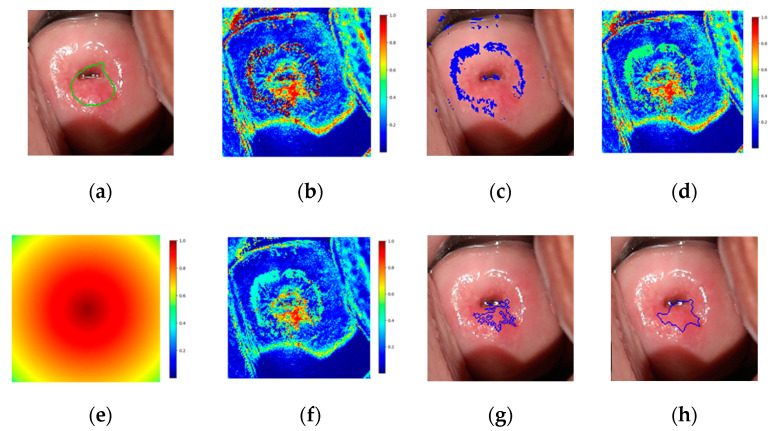
(**a**) Ground truth label drawn in the image corresponding to one minute, (**b**) probability map, (**c**) detected reflections, (**d**) probability map after identifying reflections, (**e**) distance map, (**f**) modified probability map after multiplying by the distance map, (**g**) region growing segmentation results, (**h**) resulting prediction after closing operation drawn in the image corresponding to one minute.

For the training data, the positive and negative pixels were selected, as explained in Section 2.1.1. Two additional steps were applied to the pixels selected for training. First, pixels that contain reflections were identified by thresholding the amplitude of the maximum of its time evolution. Figure 5c shows the detected reflections for a specific sequence. Second, data augmentation by scaling was applied to positive pixels, ensuring an equal number of positive and negative pixels for training.

#### 2.2.2. Neural Network Architecture

The classification was performed by using an artificial neural network (ANN), which has been proven to have the capability to model complex relationships between inputs and outputs to find patterns in data. We trained our network in a supervised manner, and we attempted to learn a function that maps an input to an output based on example input–output pairs, with the input being the intensity curve of a pixel. The neural network applied contains three layers: one input layer, a hidden layer with 15 nodes, and an output layer.

Leave-one-out cross-validation at the patient level was applied: when testing the algorithm for a specific patient, its pixels are removed from the training set and are not used for training or validation.

#### 2.2.3. Postprocessing

As an output of the ANN, the probability of each pixel of being precancerous is given, which can be combined as shown in Figure 5b, resulting in a probability map. Reflections were detected as in the preprocessing of training pixels (Figure 5c), and the probability was set to 0.5 (Figure 5d). The probability map was then multiplied by a distance map with maximum and minimum of 1 and 0.5 (Figure 5e). The distance map applied intends to emphasize that precancerous regions tend to appear in the central regions of the cervix. The final probability map is shown Figure 5f.

From the resulting probability map, region growing segmentation was applied as follows:

1. The first seed is randomly selected from the highest values of the modified probability map and added to the region.
2. The pixel’s four adjacent neighbors are separately analyzed. Any neighbor is considered to lie on the affected region if they satisfy two criteria:
  - Homogeneity criterion: the difference between the seed probability and the neighbor should be less than a fixed threshold.
  - Minimum probability criterion: the probability of the neighbor should be above a threshold.
3. The newly added pixels are compared to their own neighbors under the same criteria.
4. Steps 2 and 3 are repeated until the criteria are not met for any neighbor of the pixels lying on the region.
5. A new seed is selected such that it has the highest probability and has not been identified as part of the region before.
6. The procedure is repeated until the predefined maximum number of seeds has been distributed.

Following the region growing segmentation, a closing operation was performed, which is dilation followed by erosion with the same kernel, and the contours of the found regions were obtained. An example of the results of the region growing segmentation and closing operation is shown in Figure 5g,h, respectively. Finally, a threshold was applied to the size of the lesions detected, deciding whether they are neoplastic (positive) or not.

## 3. Results

In this section, we examine the results and performance of our classifier. Since the number of sequences is low, leave-one-out cross-validation was applied at the patient level. To guarantee class balance, the same number of negative pixels as total positive pixels available for training were randomly selected. The parameters used are specified in Table 2.

**Figure 6:**
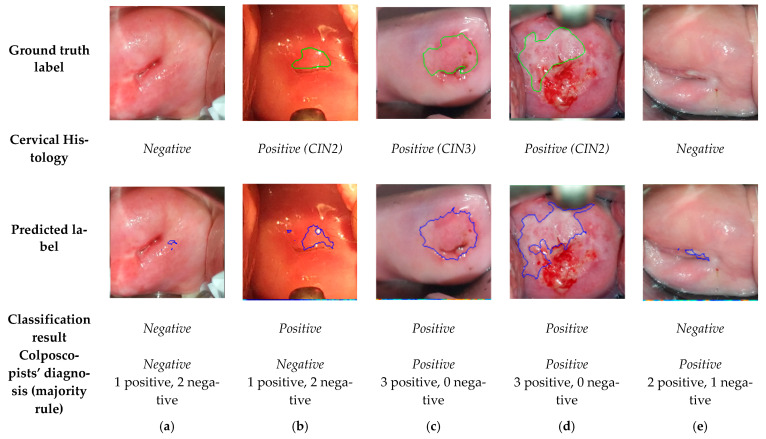
Frames corresponding to one minute after acetic acid application with the ground truth labels, histology results, predicted labels by the algorithm, resulting classification, and colposcopists’ diagnosis.

The classifier results will be compared to (i) histologic assessment and colposcopists’ diagnosis, and (ii) colposcopists’ annotations after VIA. Figure 6 shows the algorithm results for five examples of the dataset.

### 3.1. Comparision of the Final Sequence Classification, Colposcopists’ Classification, and Histology Results

In this section, the results of our model are compared to the output of the histologic assessment (negative or positive for CIN2+), considered as the reference standard. From the 44 sequences classified, 39 were classified correctly as positive or negative, giving rise to an accuracy of 0.89. The precision, sensitivity, and specificity achieved were 0.93, 0.90, and 0.87, respectively (Table 3).

The 44 sequences of the database have been shown to three colposcopists and classified as positive or negative, giving rise to the results shown in Table 3. None of the colposcopists reached a better performance than the algorithm, achieving, on average, an accuracy, precision, sensitivity, and specificity of 0.71, 0.85, 0.68, and 0.78, respectively. The Cohen’s kappa coefficient between the three colposcopists and the proposed method has been computed, which varies within the range from 0.25 to 0.62. Consequently, there is a fair to substantial agreement between the algorithm and the three expert colposcopists.

**Figure 7:**
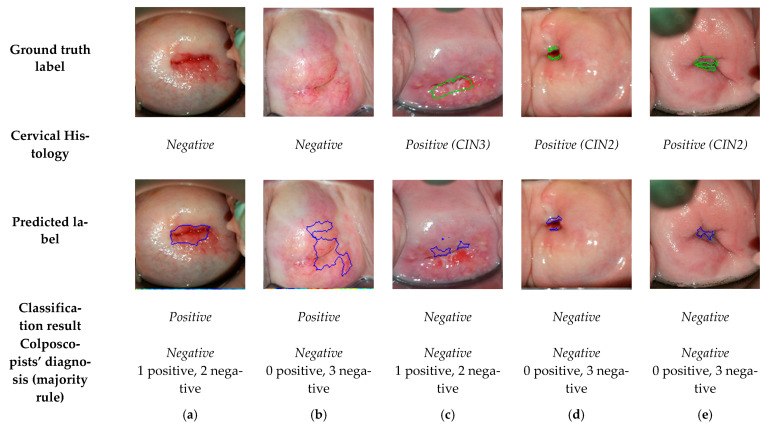
Frames corresponding to one minute after acetic acid application from dynamic images that have misclassified, with the ground truth labels, histology results, predicted labels, resulting classification, and colposcopists’ diagnosis.

The 5 sequences wrongly classified by the algorithm are shown in Figure 7. The first two are false positives: (a) an ectropion associated with inflammation and (b) an area of metaplasia. Case (a) has also been misclassified by one of the colposopists. The last three are false negative, (c), (d), and (e). For these cases, the transformation zone (TZ), i.e., the area that almost all manifestations of cervical carcinogenesis occur, is not fully visible and extends into the endocervical canal. In a case where precancerous lesions accompany TZ into the endocervical canal, visual inspection may be negative. For this reason, (c), (d), and (e) have also been misclassified by the colposcopists. Nevertheless, note that our model has found lesions similar to the ground truth in (d) and (e), but due to their small size, it has discarded them as negative.

### 3.2. Comparision of the Predicted Lesions by the Algorithm and Annotations by Colposcopists during VIA

**Figure 8:**
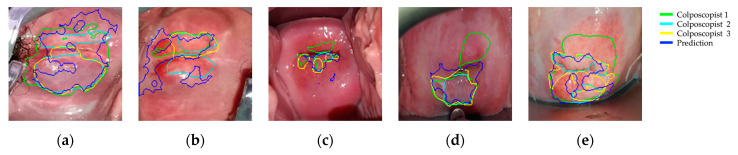
Colposcopists’ annotations and algorithm’s predicted labels drawn in one-minute frames.

VIA presents a high observer variability among colposcopists. Therefore, the annotations drawn in the last frames of our sequences are subjective, a fact highlighted in Figure 8, where the annotations given by the three colposcopists for five positive patients and the resulting predictions are represented. Note that in Figure 8a–d, colposcopists even disagree if lesions are placed in the two sides of the cervix or not, and in all cases, the annotations are significantly different.

We have extracted the positive and negative pixels for training the annotations given by the first colposcopist (represented in green) after erosion and dilation, but we have estimated the performance of the lesion positioning of our model by comparing the results to three available annotations. Note that the inaccuracy of the annotations is aggravated by the movement, especially present in the borders of the lesions.

Each preprocessed dynamic image contains 150 × 150 pixels which are independently classified. On the one hand, the corresponding annotation indicates us as to whether a pixel lies on a neoplastic lesion or not. On the other hand, the proposed method, after the region growing and closing operation, classifies every pixel as belonging to a neoplastic lesion or not. By comparing both, we can compute for each image the accuracy, precision, sensitivity, and specificity achieved by the classification of its pixels. Table 4 presents the results after averaging among images and comparing them to the three available annotations. The algorithm achieved, averaging the results for the three colposcopists’ annotations, a mean accuracy, precision, sensitivity, and specificity of 0.92, 0.53, 0.48, and 0.96, respectively. To evaluate the overlapping between the ground truth labels and the predicted ones, the Intersection over Union (IoU) has been computed, which is, on average, 0.32.

## 4. Discussion

In this study, we have developed and validated a smartphone-based solution that automatically detects cervical precancer from videos taken during VIA, intended to be used in sub-Saharan Africa as a triage method of HPV-positive women. We have measured the diagnostic accuracy of the model for detecting CIN2+ lesions and compared with the diagnostic accuracy of gynecologic oncologists. The proposed method has achieved an accuracy, sensitivity, and specificity of 0.89, 0.9, and 0.87, using histologic assessment as a reference standard.

The proposed algorithm results in a significant improvement compared to the reported sensitivities and specificities of VIA, which vary between 0.25 and 0.80, and between 0.40 and 0.90 among studies [3,4,40,41]. Furthermore, in our study, we asked three expert colposcopists blinded from the histopathologic diagnoses to classify the dynamic images from our database. The algorithm outperformed all of three of the experts, achieving a mean improvement of accuracy, sensitivity, and specificity of 17.42%, 21.84%, and 8.89%. The Cohen’s kappa coefficient, averaging among colposcopists, is 0.47, which implies that there is a moderate agreement between the proposed algorithm and the experts.

Tools using AI for cervical cancer screening have shown impressive effectiveness in automatic classification of cervical neoplastic lesions, as shown in Table 5, where some of the latest results in the literature are presented. It is important to emphasize that our algorithm presents comparable results to these state-of-the-art computer-aided diagnosis techniques, but with a much simpler setting: only VIA images are used and acquired with a smartphone, without any additional accessory. The other approaches in Table 5 require the use of colposcopes, which are rarely available in low-income countries, and some of them require a more sophisticated setup, acquiring images not only during VIA, but also during VILI and with the green lens.

Furthermore, in this work, we have been severely limited by the number of available sequences. To overcome this limitation, the inputs to the neural network are pixels’ time evolutions and leave-one-out cross-validation, at the patient level, has been applied. Nevertheless, the pixel classification approach poses several challenges. First, the selection of positive and negative pixels relies on very subjective colposcopists’ annotations and they are affected by movement. The subjectivity of the annotations has been demonstrated by comparing the annotations of the three colposcopists. We have palliated these issues by (i) applying motion compensation, (ii) selecting the pixels after eroding and dilating the annotations, and (iii) post-processing together the neural network’s output of all the pixels of one dynamic image’s pixels, ensuring homogeneity between neighboring pixels. Second, while the patient classification can be objectively evaluated by comparing it to the histological assessment, the colposcopists’ annotations are not an objective ground truth of the positions of the lesions. To have an estimation of the performance of the positioning, the predicted lesions have been compared with three colposcopists’ annotations. Third, specular reflections during recording hinder the classification of the pixels. For training, our method discards the pixels containing reflections, which are automatically detected. When predicting an image, the probability of the pixels containing reflections is set to 0.5, before starting the postprocessing of the image.

Our model failed to classify 5 sequences out of 44: in 3 of them, TZ was not fully visible and extended into the endocervical canal. The vast majority of precancers occur in the TZ. Therefore, if TZ lies in the endocervical canal, precancerous lesions may not be visible. For this reason, these three cases have been misclassified by colposcopists and by the algorithm. A cervical ectropion associated with inflammation and a case of metaplasia were misclassified as positive. Benign lesions such as inflammations, metaplasia, leukoplakia, condyloma, and CIN1 will be added as an additional class in our model in the near future. Our goal is implementing an algorithm able to distinguish between neoplastic lesions, benign lesions, and normal epithelium.

Among the different advantages of our screening tool, the minimal training required for using it and the increasing penetration rate of smartphones in LMICs make it ideal for countries with limited healthcare resources, where it can be easily integrated into a single-visit approach with the aim of reducing the preventable burden of morbidity, mortality, disability, and of reducing inequality.

Because this is a proof-of-principle investigation, it has limitations. Our selected dataset of video sequences contains a large number of positive cases, which does not correspond to the CIN2+ prevalence of a real-world setting in sub-Saharan Africa. Although these results are promising, further work is needed to improve the performance of the algorithm. First, we will increase the amount of training data and secondly, the upgraded algorithm will be validated in Cameroon in a real-life diagnosis process.

## 5. Conclusions

This work presented a smartphone-based algorithm to detect precancerous lesions from videos taken during VIA. Using the histological assessment as a gold standard, the proposed model achieved an accuracy, sensitivity, and specificity of 0.89, 0.9, and 0.87. Furthermore, the algorithm was compared to three gynecologic oncologists, outperforming all of them. The proposed model could create a new opportunity to facilitate the triage of HPV-positive women in LMICs.

## Figures and Tables

**Table 1:** Dataset specifications.

Dataset Specifications	
Number of sequences available	68
Total number of discarded sequences	24
Missing histology results and VIA annotations	2
Severe movement	16
Blurriness	2
Blood flow that prevents the visualization of the cervix tissue	3
Excess of mucus that prevents the visualization of the cervix tissue	1
Total number of sequences used	44
Number of positive sequences	29
CIN3	18
CIN2	11
Negative sequences	15
CIN1	3
Negative	12
Video length (seconds)	120
Number of frames	120
Number of selected positive pixels	21,851
Number of selected negative pixels	93,725

**Table 2:** Algorithm parameters.

Preprocessing Parameters	
Mean principal axis	[0.3609, 0.5941, 0.7074]
Discarded number of frames	First 10
Downsampling factor	0.1
Number of input features ANN	11
Range scaling coefficients used for data augmentation	[0.9, 1.15]
Reflections’ threshold	0.25
**ANN Parameters**	
Number of nodes	15
Number of hidden layers	1
**Postprocessing Parameters**	
Homogeneity criteria probability threshold	0.27
Probability threshold	0.50
Reflections’ threshold	0.25
Number of seeds	5
Closing cluster kernel	[7, 7]
Final size threshold	450

**Table 3:** Algorithm and colposcopists’ classification results based on histologic assessment.

	Algorithm	Colposcopist 1	Colposcopist 2	Colposcopist 3
True Positives	26	23	14	22
True Negatives	13	12	10	13
False Positives	2	3	5	2
False Negatives	3	6	15	7
Accuracy	0.89	0.80	0.55	0.80
Precision	0.93	0.88	0.74	0.92
Sensitivity	0.90	0.79	0.48	0.76
Specificity	0.87	0.80	0.67	0.87
Cohen’s kappa	-	0.62	0.25	0.53

**Table 4:** Results when comparing predicted lesions by the algorithm and visual inspection with acetic acid (VIA) annotations by colposcopists.

	Colposcopist 1	Colposcopist 2	Colposcopist 3
Accuracy	0.92 ± 0.10	0.92 ± 0.08	0.91 ± 0.15
Precision	0.57 ± 0.28	0.49 ± 0.29	0.55 ± 0.3
Sensitivity	0.46 ± 0.25	0.49 ± 0.29	0.5 ± 0.3
Specificity	0.97 ± 0.04	0.96 ± 0.05	0.96 ± 0.04
Intersection over Union (IoU)	0.32 ± 0.19	0.32 ± 0.20	0.33 ± 0.19

**Table 5:** Comparison of the proposed and state-of-the-art methods published between 2019 and 2021.

	Accuracy	Sensitivity	Specificity	Acquisition Device	Number of Images Captured during VIA	Additional Images Used	Number of Patients
Asiedu et al. [22]	0.8	0.81	0.786	Portable colposcope	1	VILI	134
Miyagi et al. [19]	0.82	0.80	0.88	Colposcope	1	None	330
Yue et al. [34]	0.96	0.95	0.98	Colposcope	5	VILI and green lens	679
Li et al. [33]	0.78	0.78	Not specified	Colposcope	4	Pre-acetic acid	7668
Kudva et al. [42]	0.94	0.91	0.89	Colposcope	1	None	2198
Zhang et al. [43]	0.76	0.44	0.88	Colposcope	1	None	229
Proposed algorithm	0.89	0.9	0.87	Smartphone	120	None	44

## Data Availability

The data presented in this study are available on request from the corresponding author. The data are not publicly available due to privacy restrictions.
